# Menstruation disorders in adolescents with eating disorders – target body mass index percentiles for their resolution

**DOI:** 10.1590/S1679-45082014AO2942

**Published:** 2014

**Authors:** Beatriz Vale, Sara Brito, Lígia Paulos, Pascoal Moleiro

**Affiliations:** 1 Hospital Pediátrico Carmona de Mota, Centro Hospitalar Universitário de Coimbra, Coimbra, Portugal; 2 Centro Hospitalar de Leiria, Leiria, Portugal

**Keywords:** Feeding behavior, Menstruation disturbances, Body mass index, Adolescents

## Abstract

**Objective:**

To analyse the progression of body mass index in eating disorders and to determine the percentile for establishment and resolution of the disease.

**Methods:**

A retrospective descriptive cross-sectional study. Review of clinical files of adolescents with eating disorders.

**Results:**

Of the 62 female adolescents studied with eating disorders, 51 presented with eating disorder not otherwise specified, 10 anorexia nervosa, and 1 bulimia nervosa. Twenty-one of these adolescents had menstrual disorders; in that, 14 secondary amenorrhea and 7 menstrual irregularities (6 eating disorder not otherwise specified, and 1 bulimia nervosa). In average, in anorectic adolescents, the initial body mass index was in 75th percentile; secondary amenorrhea was established 1 month after onset of the disease; minimum weight was 76.6% of ideal body mass index (at 4th percentile) at 10.2 months of disease; and resolution of amenorrhea occurred at 24 months, with average weight recovery of 93.4% of the ideal. In eating disorder not otherwise specified with menstrual disorder (n=10), the mean initial body mass index was at 85th percentile; minimal weight was in average 97.7% of the ideal value (minimum body mass index was in 52nd percentile) at 14.9 months of disease; body mass index stabilization occured at 1.6 year of disease; and mean body mass index was in 73rd percentile. Considering eating disorder not otherwise specified with secondary amenorrhea (n=4); secondary amenorrhea occurred at 4 months, with resolution at 12 months of disease (mean 65th percentile body mass index).

**Conclusion:**

One-third of the eating disorder group had menstrual disorder – two-thirds presented with amenorrhea. This study indicated that for the resolution of their menstrual disturbance the body mass index percentiles to be achieved by female adolescents with eating disorders was 25–50 in anorexia nervosa, and 50–75, in eating disorder not otherwise specified.

## RESUMO

**Objetivo:**

Analisar a progressão do índice de massa corporal nos transtornos alimentares e determinar o percentil para estabelecimento e resolução da doença.

**Métodos:**

Estudo retrospectivo descritivo, com análise dos processos de adolescentes com transtorno alimentar.

**Resultados:**

Das 62 adolescentes com distúrbio alimentar, 51 apresentavam distúrbio sem outra especificação, 10 anorexia nervosa, e uma bulimia. Vinte e uma adolescentes apresentavam distúrbios menstruais, sendo 14 amenorreia secundária, 7 irregularidades menstruais (6 distúrbio alimentar sem outra especificação e 1 bulimia). Em média, nas anoréticas: índice de massa corporal inicial - percentil 75, instalação da amenorreia secundária com um mês de doença, peso mínimo 76,6% do índice de massa corporal ideal, no percentil 4), com 10,2 meses de doença, resolução da amenorreia aos 24 meses com recuperação ponderal média de 93,4% do peso ideal. No distúrbio alimentar sem outra especificação com distúrbio menstrual (n=10), o índice de massa corporal inicial médio no percentil 85, peso mínimo, em média, 97,7% do valor de peso ideal (média no percentil 52 de índice de massa corporal), aos 14,9 meses, estabilização ponderal aos 1,6 anos, percentil médio de índice de massa corporal de 73. Na perturbação do comportamento alimentar sem outra especificação com amenorreia secundária (n=4): amenorreia secundária aos 4 meses, resolução aos 12 meses (média no percentil 65 do índice de massa corporal).

**Conclusão:**

Um terço do grupo com transtornos alimentares teve distúrbios menstruais – sendo dois terços com amenorreia secundária. Este estudo indicou que, para resolução do distúrbio menstrual, o percentil de índice de massa corporal a ser atingido na anorexia nervosa foi de 25–50 e, em casos de transtorno alimentar sem outra especificação, foi de 50–75.

## INTRODUCTION

Eating disorders (ED) in children and adolescents continue to be a serious problem and may result in premature death or life-long medical and psychosocial morbidity.^(1)^ ED are classified according to Diagnostic and Statistical Manual of Mental Disorders, 4th edition (DSM-IV), as anorexia nervosa (AN), bulimia nervosa (BN), and eating disorder not otherwise specified (EDNOS).^(2)^

ED have a peak incidence in adolescence and in females. ED diagnosis criteria cannot be fully applied to children and adolescents, making it difficult to establish the rates of ED in this population.^(3)^ EDNOS is described as the most prevalent type.^(4–6)^ In a Portuguese study of female students aged 12 to 23, EDNOS accounted for 77.4% of ED.^(6)^ The prevalence of AN in adolescents is 0.3 to 2.2%, and DSM-IV diagnostic criteria of BN are met by 0.1 to 2% of adolescents.^(3)^

The DSM-5, released in May 2013, proposes some significant changes for the diagnosis of ED, among which the removal of amenorrhea criteria from the diagnosis of AN; a separate diagnosis of binge-eating disorders will be added, which now falls under the diagnosis of EDNOS; and in BN, the number of episodes per week of binging and purging will be reduced to one. These changes in DSM 5, with less strict criteria for AN and BN and a new ED diagnosis, will reduce the prevalence of EDNOS.^(6)^

The systematic evaluation of menstrual cycle disorders in adolescence provides a window of opportunity for early diagnosis and treatment, which may have long-term adverse health consequences.^(7)^ Amenorrhea is associated with ED in 68% of cases, and there are some favoring factors, such as low body weight, excessive exercise, stress, and calorie restriction with negative energy balance.^(4,5)^ Menstrual disorders are common in adolescents, particularly in the first 2 to 3 years after menarche.^(5)^

Amenorrhea is defined as the absence of spontaneous menstruation in women during reproductive age.^(4,5)^ In adolescents, it is divided into primary and secondary amenorrhea.^(5–7)^ The former corresponds to the absence of menarche up to 15 years of age in the presence of normal secondary sexual characteristics and regular growth, or to the absence of secondary sexual characteristics in a 13-year-old female or in the presence of isolated thelarche for over 3 years.^(5)^ Secondary amenorrhea is the absence of menstruation for more than 3 months when preceded by regular menstrual cycles.^(5,7)^

There is a relationship between age of menarche and body mass index (BMI), with an earlier menarche associated with a higher BMI.^(5)^ An hypothalamic etiology is the most prevalent cause of amenorrhea in adolescence, followed by polycistic ovary syndrome and ED.^(4,5)^ Amenorrhea is responsible for significant morbidity, such as decreased bone mineral density or endometrial carcinoma, and is pathological.^(4)^ Most bone acquisition occurs during early childhood and late adolescence. The adolescent years are particularly critical, since during this period 60% of peak bone mass is acquired.^(8)^

Menstrual irregularities (MI) are defined as the presence of anovulatory cycles 3 years after menarche.^(9)^ MI in young girls is related to essential hyperandrogenism, such as polycystic ovary syndrome, and ED of the bulimic type is frequent in women with MI.^(10)^

The mechanism responsible for amenorrhea or MI in ED is not well established. Menstruation is a cyclic bleeding of the uterine mucosa, resulting from the interaction of hypothalamus-pituitary-ovarian axis hormones.^(7)^

In AN, amenorrhea is related to severe calorie restriction and subsequent suppression of the hypothalamic-pituitary axis. There are regulation changes in gonadotropin-releasing hormone (GnRH) pulsatile release, and reversal of luteinizing hormone (LH) pulsatile secretion to pre-pubertal patterns, with suppression of the pituitary production of LH and follicle-stimulating hormone (FSH). Without normal cycling of LH and FSH, the circulating level of estrogen is very low and ovulation will not occur.^(4,5)^ Approximately 20% of patients with AN develop amenorrhea before significant weight loss.^(5)^ Nutritional rehabilitation and weight recovery favor the resolution of amenorrhea.^(4,5)^

About half of adolescents suffering from BN have hypothalamic dysfunction with oligomenorrhea or MI. The latter consequence is related to nutritional restriction indexes that are not associated with low body weight.^(4)^ Although body weight remains in the normal range, amenorrhea may occur in 7 to 40% of cases.^.^ Irregular menstrual cycles (oligomenorrhea) are more frequent, in a variable proportion of 37 to 64%. The mechanism of action appears to be related to hypothalamic-pituitary dysfunction and subsequent reduced estradiol, LH, and noradrenaline levels, as well as with altered LH pulses.^(11)^

Weight recovery is a therapeutic goal in ED, but there is no consensus concerning target weight.^(12)^ According to the American Psychiatric Association (APA), the goals of nutritional rehabilitation for seriously underweight patients are to restore weight, normalize eating patterns, achieve normal perceptions of hunger and satiety, and correct biological and psychological sequelae of malnutrition.

Unlike adults, for whom values such as ideal body weight or premorbid weight are used,^(9,10)^ there is no consensus about the precise target weight to be reached in adolescents with ED. At this young age, genetic and environmental factors, puberty development, and continuous growth in various anthropometric parameters (best reflected by percentiles – P) make clinical goals distinct in this population. There is a lack of consensus as to how to determine the treatment goal weight in the growing adolescent, when both height and weight are changing as part of normal development.^(12)^ For patients aged 20 years and younger, the individually appropriate range for expected weight and the goals for weight and height should be determined considering measurements and clinical factors, including current weight, bone age estimated from wrist X-rays and normograms, and menstrual history – in adolescents with secondary amenorrhea, we observe mid-parental heights, assessments of skeletal frame, and benchmarks from Centers for Disease Control and Prevention (CDC) growth charts.^(13)^

Menstrual regularization is a biological health indicator. Some studies have associated anthropometry of menstrual regularization with the goal weight in AN.^(12)^ This data may possibly be extended to all types of ED. In pediatric clinical practice, evaluation of growth is based on P growth charts. Growth charts consist of a series of P curves that illustrate the distribution of selected body measurements in children. For children, BMI is age- and gender-specific, and nutritional status is identified based on P.^(14)^

## OBJECTIVE

The purpose of this study was to analyse the progression of body mass index percentiles, which are the most reliable anthropometric tool, of female adolescents with eating disorders and to determine this index P during eating disorders progression in the establishment and resolution of menstrual disorders.

## METHODS

The authors conducted a retrospective, descriptive, cross-sectional study, with review of the clinical records of female adolescents followed for ED in an outpatient adolescent clinic from August 2005 to August 2010.

The study included adolescents with ED diagnosed according to DSM-IV criteria (Chart 1, 2, and 3) with menstrual disorders (secondary amenorrhea and MI). Secondary amenorrhea was defined as the absence of menstruation for more than 3 months when preceded by regular menstrual cycles, and MI consisted of the presence of anovulatory cycles 3 years after menarche.

**Chart 1:** Diagnostic criteria for anorexia nervosa^(2)^

DSM-IV – Anorexia nervosa criteria
– Refusal to maintain body weight at or above a minimally normal weight for age and height: weight loss leading to maintenance of body weight less than 85% of that expected, or failure to make expected weight gain during period of growth, leading to body weight less than 85% of that expected – Intense fear of gaining weight or becoming fat, even though underweight – Disorder in the way in which one's body weight or shape is experienced, undue influence of body shape on self-evaluation, or denial of the seriousness of the current low body weight – Post-menarcheal females, amenorrhea, i.e., the absence of at least three consecutive menstrual cycles – DSM-IV: anorexia nervosa – Types Restricting Type: weight loss after diet, fasting, or excessive exercise. Binge-Eating/Purging Type: weight loss with regular binge eating, purging (vomiting, laxatives, enemas, diuretics), or both

DSM IV: Diagnostic and Statistical Manual of Mental Disorders IV.

**Chart 2:** Diagnostic criteria for bulimia nervosa^(2)^

DSM-IV – Bulimia nervosa criteria
– Recurrent episodes of binge eating. An episode of binge eating is characterized by both of the following: – Eating, in a discrete period of time (e.g., within any 2-hour period), an amount of food that is definitely larger than most people would eat during a similar period of time and under similar circumstances – A sense of lack of control over eating during the episode (e.g., a feeling that one cannot stop eating or control what or how much one is eating) – Recurrent inappropriate compensatory behaviour in order to prevent weight gain, such as self-induced vomiting, misuse of laxatives, diuretics, enemas, or other medications; fasting, or excessive exercise – Binge eating and inappropriate compensatory behaviours occur, on average, at least twice a week for 3 months – Self-evaluation is unduly influenced by body shape and weight – The disorder does not occur exclusively during episodes of anorexia nervosa – DSM-IV: bulimia nervosa – Types Purging type: regular self-induced vomiting, misuse of laxatives, diuretics, enemas, or other medications Non-purging type: exercise and fasting to compensate

DSM IV: Diagnostic and Statistical Manual of Mental Disorders IV.

**Chart 3:** Diagnostic criteria for eating disorder not otherwise specified^(2)^

DSM-IV – Eating disorder not otherwise specified
Criteria
– All diagnostic criteria for anorexia nervosa are met, but the menstrual cycle is normal – All diagnostic criteria for anorexia nervosa are met, but weight is normal for height and age even after considerable weight loss – All diagnostic criteria for bulimia nervosa are met, but the frequency of binges is less than twice weekly and for a duration of less than 3 months – There are recurring efforts to compensate (such as self-induced vomiting) for eating only small amounts of food, but body weight is normal for height and age – Regularly chewing and spitting out large quantities of food without swallowing – Binge-eating disorder: regular episodes of binge eating, but with no recurring efforts to compensate, such as purging or excessive exercise

DSM IV: Diagnostic and Statistical Manual of Mental Disorders IV.

Data collection was based on review of the adolescents' clinical records, as long as there was no loss of information or refusal to participate. Confidenciality, data anonymity, and professional secrecy were preserved and there were no ethical issues concerned.

The variables analyzed were age, type of ED, menstrual disorders, and BMI percentile at four disease progression moments: beginning of disease, establishment and resolution of menstrual disorders, and the lowest BMI.

The exact BMI percentile (P) was calculated using the Child and Teen BMI calculator, available at http://apps.nccd.cdc.gov/dnpabmi. BMI was calculated using the formula: weight/height^2^.

Overweight was defined as BMI at or above the P85 and lower than the P95 for children of the same age and genre, and obesity as BMI at or above the P95 for children of the same age and genre, according to 2000 CDC growth charts.^(15)^ Disease onset corresponded to the patient's initial signs/symptoms of ED referred by the adolescent and/or relatives.

Statistical data analysis was performed using the Excel for Windows 2007^®^ program.

The study was approved by *Centro Hospital de Leiria* review board, from Leiria, Portugal.

## RESULTS

From August 2005 to August 2010, 62 female adolescents were observed in the outpatient adolescent medical clinic for ED: 51 with EDNOS (82.2%), 10 with AN (16.1%), and 1 with BN (1.7%). Among the adolescents with ED, 21 had menstrual disorders (34%). As a diagnostic criterion for AN, amenorrhea was present in 100% in this group. In the EDNOS group, menstrual disorders corresponded to four secondary amenorrhea cases and six MI, with a global frequency of 20%. Patients with BN were excluded from the study.

Age distribution in the 20 girls with ED and menstrual disorders ranged from 12 to 17 years, with a higher frequency at 13 and 14 years, with 5 and 6 cases, respectively. The mean age was 14.8 years, slightly lower in AN (14.4 years) than in EDNOS (15.3 years). When grouping according to the type of menstrual disorder, we observed that the mean age at establishment of secondary amenorrhea and at AN diagnosis was similar (14.6 years) to that of the establishment of MI and at EDNOS diagnosis (15.1 years).

The analysis of anthropometric data during disease progression showed that 33% of adolescents with AN (n=10) were overweight or obese at the onset of disease (n=6/10; data of four of the patients were not available in the clinical files), with a mean BMI in the P75. The minimum BMI was in the P4 (four patients with a P<1) after 10.2 months of disease, with a 29% loss of the initial weight. This reflected a mean of 18.2kg weight loss, ranging from a minimum of 12.5kg to a maximum of 28.6kg (Table 1).

**Table 1:** Anthropometric evolutive data in the anorexia nervosa group (n=10)

Anorexia nervosa	Disease onset	Minimum value	Amenorrhea	
			Onset	Resolution
BMI percentile	75	4	16	33
Disease duration, months		10.2	1	24

BMI: body mass index.

In adolescents with AN, secondary amenorrhea was established on average after 1 month of disease with a BMI in the P16, corresponding to a 22% loss of the initial weight. At this stage, no one was overweight or obese. On average, secondary amenorrhea resolution occurred at 24 months of disease, with a BMI in the P33, corresponding to 92.6% of the initial weight. Four adolescents with AN remained amenorrheic at the end of the study.

Adolescents with EDNOS, at the onset of the disease, had a mean BMI in the P85 (MI in P87 and secondary amenorrhea in P84). Overweight and obesity were present in 55%. On average, the minimum BMI was in the P53 at 14.9 months of disease.

Secondary amenorrhea in adolescents with EDNOS was established at 4 months of disease, with a mean BMI in the P55 and a weight 15% lower than the initial value. Secondary amenorrhea resolution occurred, on average, in the P65 of BMI, at 12 months of disease progression, with 80% of the initial weight. Minimum weight was in the P43 at 11 months of disease, 19% less than the initial weight.

Addressing secondary amenorrhea as a group (including secondary amenorrhea in AN and EDNOS), it was established on average at 2 months of disease, with a mean BMI in the P29 and weight 19% lower than initially. Secondary amenorrhea resolution ocurred in the P47 of BMI at 19 months of disease, with 83% of the initial weight. Minimum weight in secondary amenorrhea was in the P16 at 10 months of disease, corresponding to 25% less than the initial weight.

Regarding MI (exclusively in EDNOS), initial BMI was in the P87 with a minimum BMI during disease progression in the P60 at 11.3 months of disease. The resolution of MI could not be assessed through clinical files. In this group, weight stabilization occurred in the P73 of BMI, at 1.6 year of disease (Table 2).

**Table 2:** Anthropometric evolutive data regarding eating disorders of the not otherwise specified group (n=51)

Eating disorder not otherwise specified	Disease onset	Minimum value	Amenorrhea	
			Onset	Resolution
BMI percentile	85	53	55	65
	(MI: 87)	(MI: 60)		
	(SA: 84)	(SA: 45)		
Disease duration, months	-	14.9	4	12

BMI: body mass index; MI: menstrual irregularities; SA: secondary amenorrhea.

## DISCUSSION

ED is a group of diseases with increasing incidence. Their possible short- and long-term comorbidities impair adolescent Quality of Life.^(4,16)^

In agreement with published literature, EDNOS represented the most prevalent subtype of ED, with rates similar to those of other studies.^(6)^ As in other published data, AN was more frequent than BN, but, in this study, the proportion was ten times greater when compared to other studies.^(3,6)^ Adolescent age range is supported by literature data, although a higher prevalence of ED during late adolescence has been described.^(16–18)^

A past personal history of obesity or overweight was present in one third of the studied population, with the highest incidence in the EDNOS group. This has been defined as a risk factor for ED development.^(17–19)^

Given that about one-third of the adolescents with ED studied suffered from menstrual disorders, this comorbidity is not negligible. As shown in this study, menstrual disorders had an early onset in ED progression. Secondary amenorrhea was established at 1 month of disease in AN and 4 months in EDNOS. Age distribution in ED girls who also had menstrual disorders ranged from 12 to 17 years, with a mean age of 14.8 years. Hence, this diagnosis should be suspected even at earlier ages.

In this adolescent population, significant weight loss rates were recorded, occurring earlier in the AN group. In AN, minimum mean BMI was in the P4 *versus* the P53 in EDNOS, and in contrast with the initial mean BMI in the P75 and P85, respectively.

Menstrual disorders were diagnosed in a third of the adolescents studied and persisted for a long period of time. In AN, the resolution of amenorrhea occurred on average 2 years after the onset of disease, with a mean BMI in the P33, and in EDNOS after 1 year of disease, in the P65 BMI, on average.

Finally, in this study secondary amenorrhea in anorectic adolescents was established in the P16 BMI, and its resolution occurred when the P33 BMI was achieved. In EDNOS, most adolescents were overweight (55%) at the onset of the disease and secondary amenorrhea was established in the P55 BMI, resolving when BMI stabilized in the P65.

This analysis indicates the P25 to P50 BMI in AN and P50 to P75 BMI in EDNOS as the approximate BMI percentiles at the resolution of menstrual disorders in female adolescents during the course of their ED. This fact is attributed to the previously mentioned information, which highlights the regularization of menstrual disorders as a probable indicator of disease stabilization.

Considering the changes for the diagnosis of ED in DSM-5, we analyzed our data considering secondary amenorrhea as a group, regardless of the ED diagnosis. Secondary amenorrhea was established at 2 months of disease, with a mean BMI in P29 and amenorrhea resolution ocurred in P47 of BMI, at 19 months of disease.

There are few studies concerning the association of anthropometric data with the resolution of menstrual disorders in ED, and these relate almost exclusively to AN. In comparison, it is noticeable that current results are similar to the ones published, particularly the BMI percentile at the moment of resolution of amenorrhea.^(12)^

The limitations of this study include the fact that data were collected retrospectively, the sample size was small, and analysis of some variables that could have a role in the determination of BMI at the resumption of menses, such as psychological issues or exercise, were not considered. In addition, it would have been valuable to compare menstrual disorders in the studied population to a control group of adolescents with the same ages and without ED, so as to support more accurate conclusions.

## CONCLUSION

The advantages of the present study include the fact that it suggests the target body mass index percentile to achieve in the resolution of menstrual disorders, a good prognosis indicator during treatment of eating disorders both for anorexia nervosa and eating disorder not otherwise specified – while other studies only approach anorexia nervosa. In this study, considering the adolescents with secondary amenorrhea as an isolated group, a target body mass index percentile, not previously mentioned in literature, was proposed for the resolution of this menstrual disorder. Based on the forthcoming changes and less strict criteria in the diagnosis of eating disorders with the publication of the updated DSM-5 classification, excluding secondary amenorrhea as a definite criteria for anorexia nervosa and replacing eating disorder not otherwise specified by the new entity “binge eating disorders”, the overall prevalence of these disorders will suffer modifications in the future.

Healthcare professionals give an essential contribution to the early detection of eating disorders in the pediatric population, through screening of suspicious signs and symptoms during routine clinical consultations, with appropriate periodic anthropometric evaluations and the systematic evaluation of menstrual cycle disorders.

## References

[1] Rome ES, Ammerman S, Rosen DS, Keller RJ, Lock J, Mammel KA (2003). Children and adolescents with eating disorders: the state of the art. Pediatrics.

[2] American Psychiatric Association (APA) (1994). Diagnostic and statistical manual of mental disorders.

[3] Portela de Santana ML, da Costa Ribeiro H, Mora Giral M, Raich RM (2012). La epidemiología y los factores de riesgo de los trastornos alimentariosen la adolescencia; una revisión. NutrHosp.

[4] Seidenfeld ME, Rickert VI (2001). Impact of anorexia, bulimia and obesity on the gynecologic health of adolescents. Am Fam Physician.

[5] Golden NH, Carlson JL (2008). The pathophysiology of amenorrhea in the adolescent. Ann N Y Acad Sci.

[6] Keel PK, Brown TA, Holm-Denoma J, Bodell LP (2011). Comparison of DSM-IV versus proposed DSM-5 diagnostic criteria for eating disorders: reduction of eating disorder not otherwise specified and validity. Int J Eat Disord.

[7] Machado PP, Machado BC, Gonçalves S, Hoek HW (2007). The prevalence of eating disorders not otherwise specified. Int J Eat Disord.

[8] Popat VB, Prodanov T, Calis KA, Nelson LM (2008). The menstrual cycle: a biological marker of general health in adolescents. Ann N Y Acad Sci.

[9] Golden NH, Jacobson MS, Schebendach J, Solanto MV, Hertz SM, Shenker IR (1997). Resumption of menses in anorexia nervosa. Arch Pediatr Adolesc Med.

[10] Montoya JS, Cabezza AH, Rojas OF, Navarrete RC, Keever MA (2012). Menstrual disorders in adolescents. Bol Med Hosp Infant Mex.

[11] Mitan LA (2004). Menstrual dysfunction in anorexia nervosa. J Pediatr Adolesc Gynecol.

[12] Wiksten-Almströmer M, Hirschberg AL, Hagenfeldt K (2008). Prospective follow-up of menstrual disorders in adolescence and prognostic factors. Acta Obstet Gynecol Scand.

[13] Golden NH, Jacobson MS, Sterling WM, Hertz S (2008). Treatment goal weight in adolescents with anorexia nervosa: use of BMI percentiles. Int J Eat Disord.

[14] (2000). Practice guideline for the treatment of patients with eating disorders (revision). American Psychiatric Association Work Group on Eating Disorders. Am J Psychiatry.

[15] Centers for Disease Control and Prevention, National Center for Health Statistics (2010). Growth Charts [Internet].

[16] Barlow SE (2007). Expert Committee. Expert committee recommendations regarding the prevention, assessment, and treatment of child and adolescent overweight and obesity: summary report. Pediatrics.

[17] Bacalhau S, Moleiro P (2010). Perturbações do comportamento alimentar em adolescentes - o que procurar?. Acta Med Port.

[18] Genndall KA, Bulik CM, Joyce PR, McIntosh VV, Carter FA (2000). Menstrual cycle irregularity in bulimia nervosa. Associated factors and changes with treatment. J Psychosom Res.

[19] Copeland PM, Sacks NR, Herzog DB (1995). Longitudinal follow-up of amenorrhea in eating disorders. Psychosom Med.

